# Magnesium-Molybate Compounds as Matrix for ^99^Mo/^99m^Tc Generators

**DOI:** 10.3390/ph4020215

**Published:** 2011-01-25

**Authors:** Fabiola Monroy-Guzman, Thania Susana Jimenez Martinez, Humberto Arriola, Luis Carlos Longoria Gandara

**Affiliations:** 1 National Institute of Nuclear Research (ININ) Carretera Mexico-Toluca, 52750, Mexico; 2 Faculty of Chemistry, National University of Mexico, Coyoacan 04510, Mexico; E-Mail: has@servidor.unam.mx (H.A.)

**Keywords:** ^99^Mo/^99m^Tc generator, magnesium molybdates

## Abstract

This work reports the preparation of a ^99m^Tc generator based on conversion of ^99^Mo produced by neutron irradiation, into insoluble magnesium ^99^Mo-molybdates compounds as matrix. The effect of magnesium salt types and concentration, Mg:Mo molar ratios, pH of molybdate solutions, eluate volume as well as the addition order of molybdate and magnesium solutions' influences on the final ^99m^Tc were evaluated. Polymetalates and polymolybdates salts either crystallized or amorphous were obtained depending on the magnesium salt and Mg:Mo molar ratio used in matrix preparation. ^99^Mo/^99m^Tc generator production based on magnesium-^99^Mo molybdate compounds allow reduction of preparation time and eliminates the use of specialized installations. The best generator performances were attained using matrices prepared from 0.1 mol/L MgCl_2_·6H_2_O solutions, ammonium molybdate solutions at pH 7 and at a Mg:Mo molar ratio of 1:1.

## 1. Introduction

Technetium-99m (^99m^Tc) is used for more than two thirds of nuclear imaging techniques because of its short 6.02 h half-life, simple decay scheme (a single 141 KeV photon), minimum whole-body dose, versatile chemistry, and availability from the ^99^Mo/^99m^Tc generator [1-2]. This system is based on adsorption of ^99^Mo on an alumina column where ^99m^Tc formed from decay of the ^99^Mo is periodically eluted from the column using physiological saline, as sodium pertechnetate (Na^99m^TcO_4_) while ^99^MoO_4_^2−^ remains attached to alumina. The limited loading capacity of alumina for molybdenum (2 mg Mo/g alumina) forces the use of a uranium fission product with a high specific activity, ^99^Mo (10^5^ Ci/g Mo) [3], thus requiring sophisticated separation processing infrastructure and disposal of large amounts of radioactive wastes [4-5]. To avoid this, alternative methods of ^99^Mo/^99m^Tc generator production have been investigated using low and medium specific activity ^99^Mo, produced from (n,γ) nuclear reaction with natural Mo (activation method) and directly converted into insoluble substrates that can be eluted in a column. ^99^Mo/^99m^Tc generator based on heteropolyanions such as zirconium molybdate, titanium molybdate, molybdocerates, *etc.*, [6-12] have been developed by some laboratories around the world. This is due to the molybdates' matrix capacity to incorporate up to 30% in weight of ^99^Mo [13] compared to 0.2% in traditional alumina based generators. Although these generators has opened a way of making column type ^99m^Tc generator even using low and medium specific activity ^99^Mo, the handling problems (precipitation, filtration drying, fragmentation, *etc.*) still exist because these ^99^Mo-molybdates are mostly synthesized from ^99^Mo, requiring sophisticated remote handling facilities and at least 6 h processing time [12,14]. To simplify the production process of these systems, we propose preparing ^99^Mo/^99m^Tc generators based on magnesium ^99^Mo-molybdate compounds by synthesizing magnesium molybdate compounds, followed by irradiation. This approach has three advantages: (1) it eliminates the use of specialized installations for molybdates synthesis; (2) it reduces ^99^Mo/^99m^Tc generator preparation time and (3) it minimizes radiological contributions at ^99m^Tc eluats due to the only radioisotope produced for the manganesium (^24^Mg) during magnesium molybdate compound irradiation which has a short half life: 9.46 min.

Systematic studies on ^99^Mo/^99m^Tc generators based on magnesium ^99^Mo-molybdate compounds were performed. The effect of six parameters on the ^99^Mo/^99m^Tc generator performance were evaluated: magnesium concentration and salt type, Mg:Mo molar ratios, molybdates solutions and precipitated pH, and addition order of molybdate and magnesium solutions. The physical-chemical properties of magnesium molybdate compounds were also determined to relate their properties with generator performance.

## 2. Results and Discussion

### 2.1. Performances of ^99^Mo/^99m^Tc Generators Based on Magnesium ^99^Mo-Molybdate Compounds

Table 1 shows the performances of the ^99^Mo/^99m^Tc generators based on magnesium ^99^Mo-molybdate compounds prepared in this research. Results are divided into three series, in line with the type of magnesium salts used in the generator preparation: magnesium chloride hexahydrate (series A), magnesium nitrate hexahydrate (series B) and magnesium sulfate hexahydrate (series C). The generator performances were compared with those advised by the Pharmacopoeia for the ^99m^Tc eluates used with medical purposes: ^99^Mo breakthrough less than 0.015%, a minimum percentage of 95% for the radiochemical purity, a chemical purity less than 10 ppm for aluminium and pH values between 4.5 and 7.5 [15].

**Figure 1:**
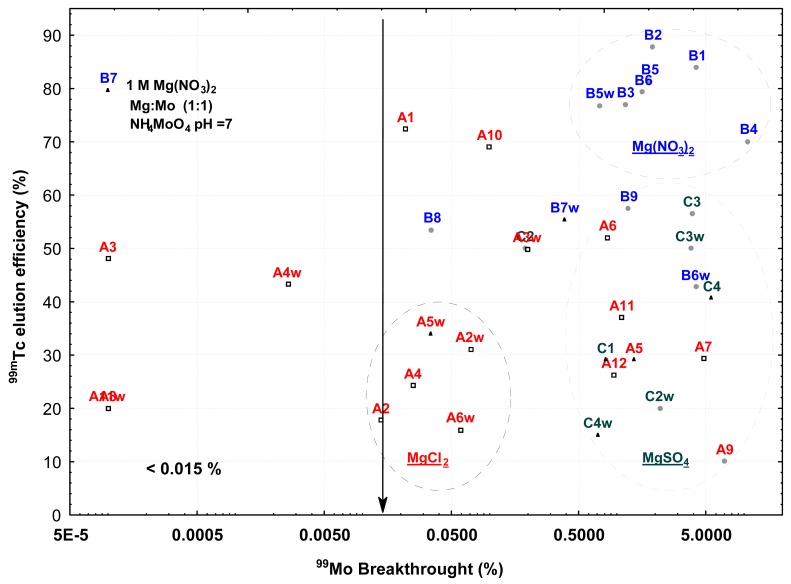

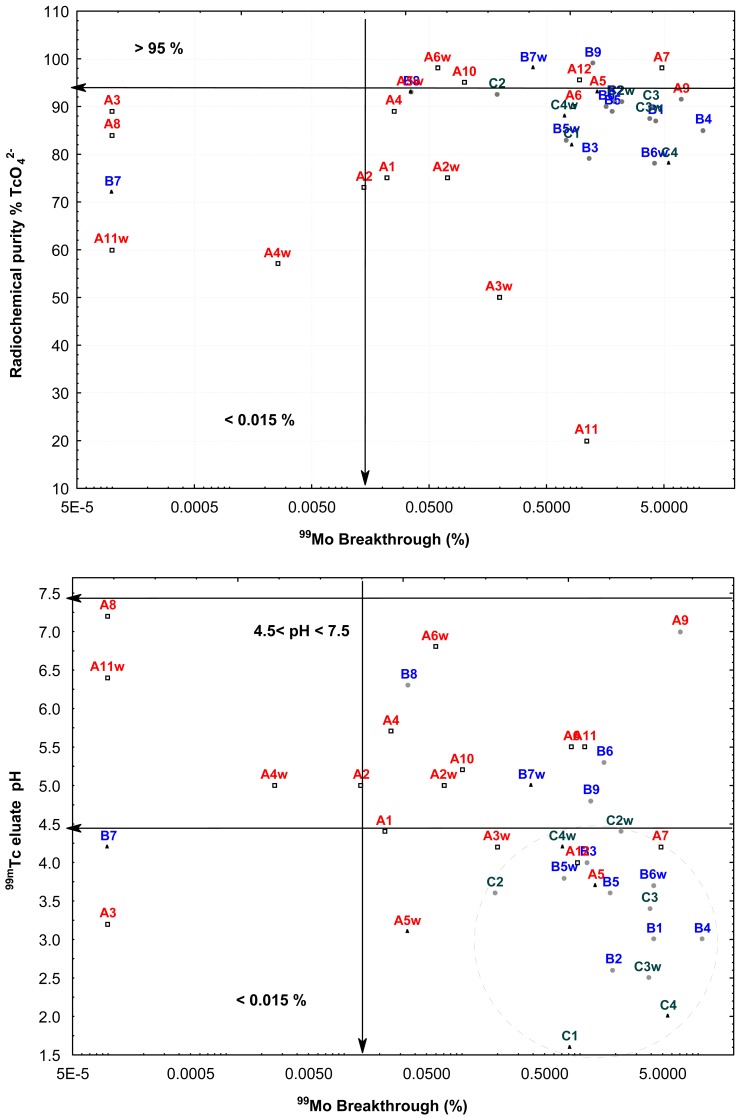
Distribution of magnesium ^99^Mo-molybdate generator performances (^99m^Tc eluate efficiency and pH, radiochemical purity) in function of ^99^Mo breakthough.

^99^Mo breakthrough percentages of less than 0.015% were only obtained in the matrices prepared from: (a) 0.5 mol/L MgCl_2_·6H_2_O (series A) and b) 1 mol/L MgNO_3_·6H_2_O solutions (series B) using ammonium molybdate solutions at pH of 7 and a Mg:Mo molar ratio of 1:2 (Figure 1). However, ^99m^Tc elution efficiencies of theses generators were less than 48%, except for the matrix B7. On the other hand, the highest elution efficiencies (>70%) were obtained in the generators prepared from 0.1 mol/L Mg(NO_3_)_2_·6H_2_O solutions, at a Mg:Mo molar ratio of 0.2:1 and ammonium molybdate solutions at pH values between 4.5 and 10, however under these conditions, ^99^Mo breakthrough of the eluates were more than 0.7%, apart from the matrix B7. ^99m^Tc eluates of the matrices prepared preferably with Mg:Mo molar ratio of 2:1 presented radiochemical purity of more than 95% and, in general, those made from MgCl_2_·6H_2_O solutions which satisfy the eluate pH values fixed by the Pharmacopoeia: between 4.5 and 7.5, while the eluate pH values obtained from the matrices formed with MgSO_4_·6H_2_O were the more acid, between 1 and 3. The average elution volume of all the generators studied ranged between 2 and 3.5 mL and all ^99m^Tc eluates had an Al content of less than 10 ppm. It is important to note that a high ^99^Mo breakthrough percentage in the eluates entails the presence of Mg^2+^ in solution.

The Mo and Mg content in the generators is directly connected with: the Mg:Mo molar ratio, the type and concentration of magnesium salt used during matrix synthesis and matrix washing before irradiation. Thus the highest (75-50%) and lowest (18–7%) Mo percentages, and *vice versa* for Mg content, were recorded in the washed matrices and those prepared from MgSO_4_·6H_2_O solutions at Mg:Mo molar ratio of 2:1 respectively.

Matrix washing caused a decrease of the ^99m^Tc elution efficiencies and Mg percentage in the matrix, while an increase in magnesium salt concentration (series C) induced a drop in the ^99m^Tc elution efficiency and acidification of ^99m^Tc eluates. The addition order of magnesium salt and ammonium molybdate solutions, and ammonium molybdate pH in the matrix process preparation (series B) did not cause meaningful changes in ^99^Mo/^99m^Tc generator performance.

^99m^Tc eluates produced by the generators prepared from MgCl_2_·6H_2_O solutions (series A) mostly attained the pH values established by the Pharmacopoeia: between 4.5 and 7.5, while higher acid eluates were obtained in the matrices synthesized from MgSO_4_·6H_2_O solutions. When the Mg proportion was higher than Mo in the Mg:Mo molar ratio, ^99^Mo breakthrough percentage increased in the ^99m^Tc eluates and the Mo percentages in the matrix decreased. All ^99m^Tc eluates of series A were colorless, those prepared from MgNO_3_·6H_2_O solutions at pH 10 or adding the ammonium molybdate solutions to magnesium salts (series B) presented a yellow coloration, while some of series C eluates showed a yellow coloring or a blue precipitate, in fact only the ^99m^Tc eluates obtained from generator prepared with 0.1 mol/L MgSO_4_·6H_2_O solutions were colorless.

### 2.2. Characterization of Magnesium Molybdate Compounds

**Figure 2:**
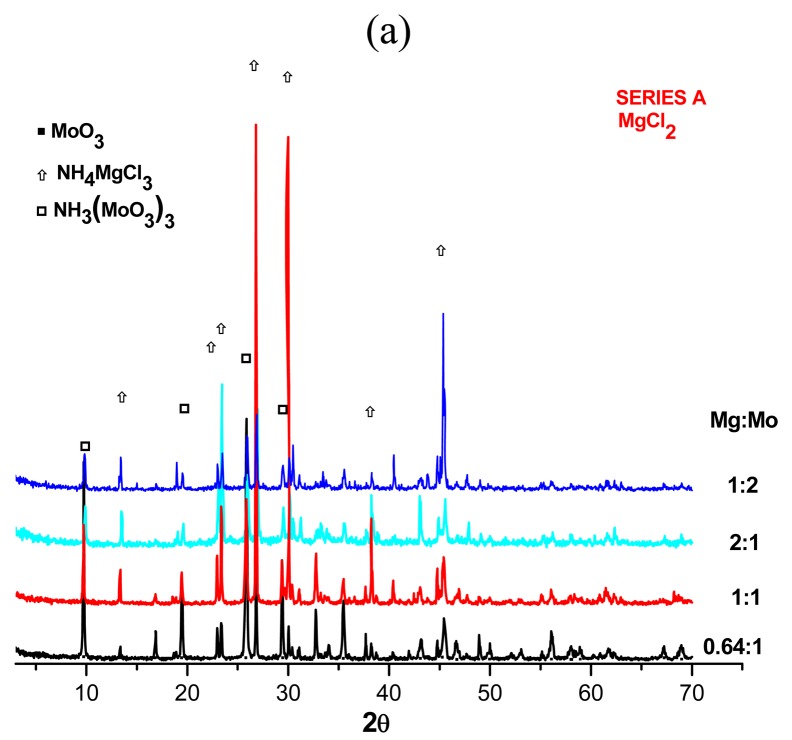

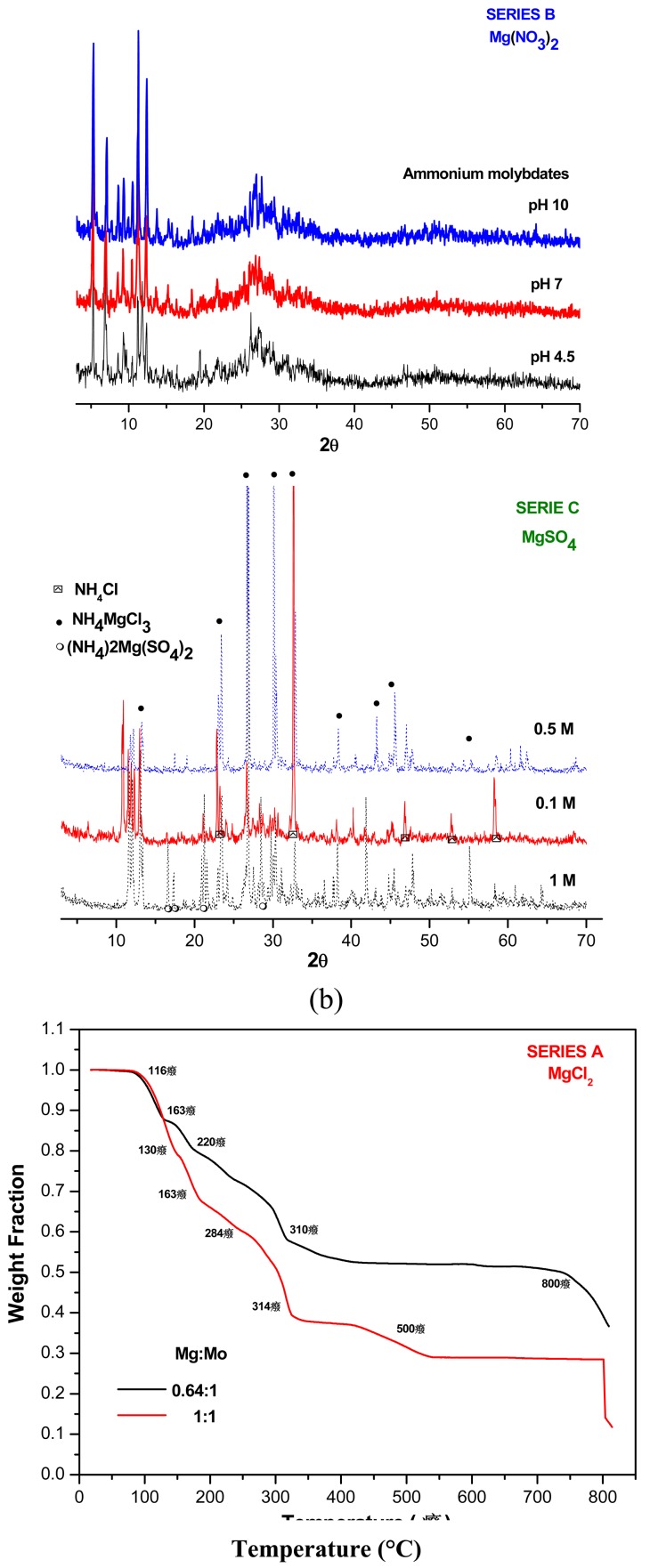

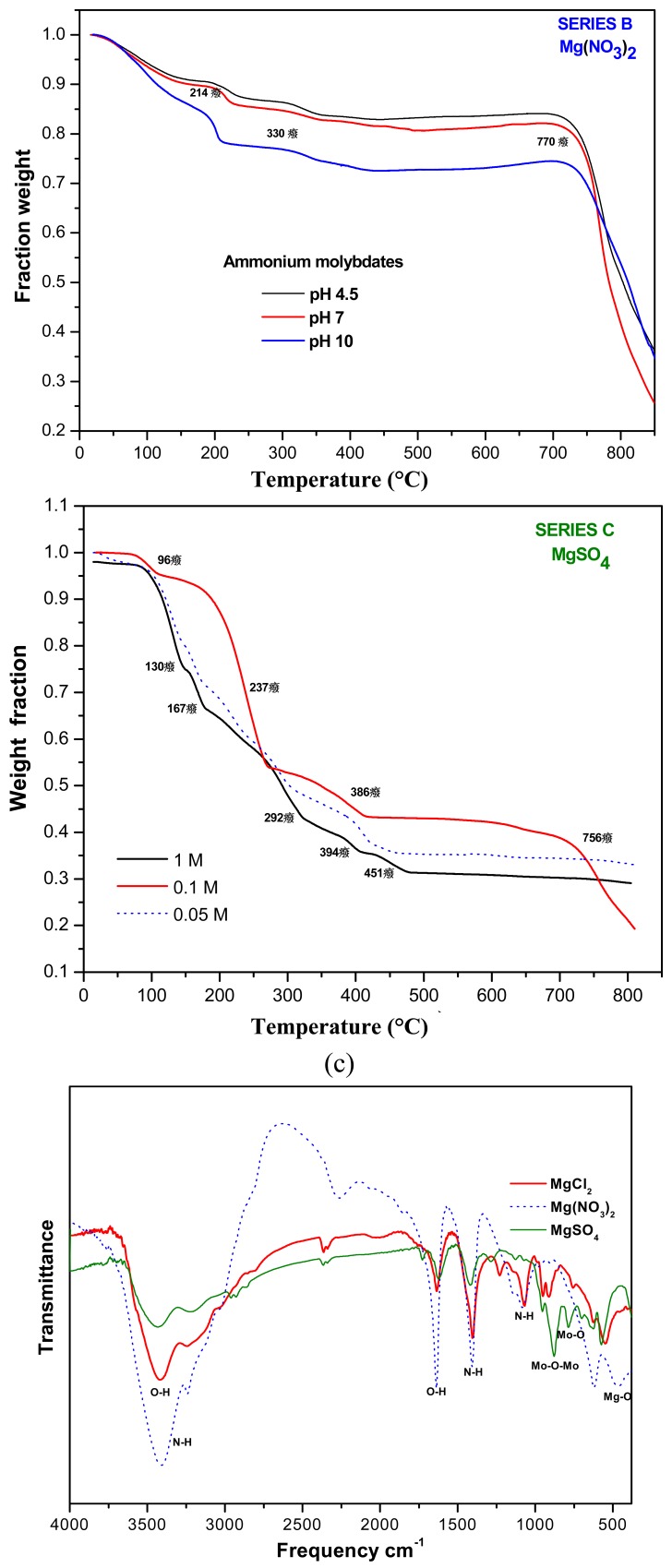
Magnesium salt effect used in the preparation of magnesium-molybdates in (a) X-ray diffraction patters, (b) thermograms and (c) infrared spectra.

Crystalline phases identified by XRD (see Table 1 and Figure 2a) showed that the type of magnesium salt used in preparing generator matrices determines their chemical composition. In accordance with these data, Mg-Mo compounds prepared from MgCl_2_·6H_2_O, Mg(NO_3_)_2_·6H_2_O and MgSO_4_·6H_2_O solutions are mainly constituted of: (a) NH_4_MgCl_3_·6H_2_O, MoO_3_ and NH_3_(MoO_3_)_3_; (b) amorphous compounds and unidentified crystalline phases and (c) NH_4_MgCl_3_·6H_2_O, (NH_4_)_2_Mg(SO_4_)_2_·6H_2_O and amorphous compounds, respectively. These results are congruent with the thermogravimetric and infrared spectra shown in Figures 2b which present a characteristic pattern for each magnesium salt used. The thermal decomposition step multiples of different matrices prove the presence of compound mixtures. In series A, it is possible to identify five main causes for weight-loss, firstly water elimination of ammonium magnesium chloride hydrate (∼116 °C), later the transformation of NH_4_MgCl_3_ in Mg(OH)Cl and NH_4_Cl (160–170 °C), after NH_4_Cl decomposition (∼220 °C), the formation of MgO from Mg(OH)Cl (350–550 °C) and finally the decomposition of MoO_3_ (770–800 °C) [8,16]. Three weight-losses are evident in the series B at 214, 330 y 770 °C which could be derived from NH_3_Cl, Mg(OH) and MoO_3_ decomposition respectively considering that amorphous materials and the unidentified phases (Figure 2b) are constituted by Mo, Mg, NO_3_^−^, NH_3_ and Cl and making an analogy with the compounds formed in series A. In the case of series C, the weight-loss is fixed by the NH_4_MgCl_3_·6H_2_O, (NH_4_)_2_Mg(SO_4_)_2_·6H_2_O and amorphous molybdenum compound decomposition (see Table 1 and Figure 2b): dehydratation (96–130 °C), elimination of [NH_4_^+^] in NH_4_MgCl_3_ (167 °C) and (NH_4_)_2_Mg(SO_4_)_2_ (451 °C), decomposition of NH_4_Cl (∼237 °C), formation of MgO (300–400 °C) and decomposition of MoO_3_ (756 °C) [16].

The effect of magnesium salt on forming different magnesium-molybdenum compounds during generator matrices preparation was also demonstrated by infrared analysis shown in Figure 2c. The matrix spectra prepared using MgCl_2_*6H_2_O, Mg(NO_3_)_2_*6H_2_O and MgSO_4_*6H_2_O solutions have similar troughs in the 3500-1200 cm^−1^ region, but with differences in band intensities.

In this region, ammonium and water displays strong broad N-H and O-H stretching bands between 3500 and 3300 cm^−1^ and bands at 1405 and 1640 cm^−1^ respectively [8,17-18]; while significant differences in intensities and wavenumber values and of each matrix were noted in fingerprint region (1200–400 cm^−1^). All matrices spectra exhibit characteristic absorption bands of Mo-O-Mo vibration at 960, 910 cm^−1^ and N-H bonds at 1075 and 1220 cm^−1^ as well as a band at 620 cm^−1^ possibly originated from Mg vibrations [8,18-19]. Only the matrix prepared from Mg(NO_3_)_2_*6H_2_O presented broad bands at around 470 cm^−1^ attributed to Mg-O bonds [19], and that from MgSO_4_*6H_2_O showed characteristic bands assigned to [SO_4_^2−^] (628, 700, 1075, 1130, 1287 cm^−1^) and Mo-O vibrations at 792 and 880 cm^−1^ whereas that from MgCl_2_*6H_2_O presented peaks 760 and 545 cm^−1^ assigned to the Mo-O vibrations and possibly to Mg-Cl bonds respectively [8,18-19].

**Figure 3:**
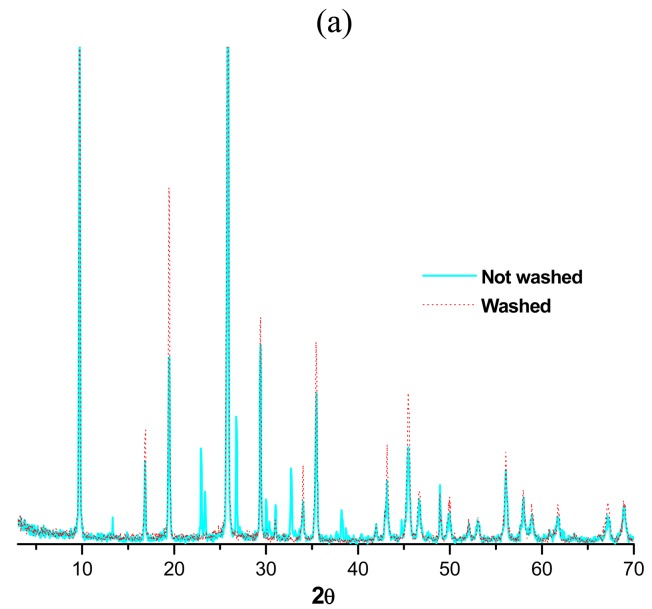

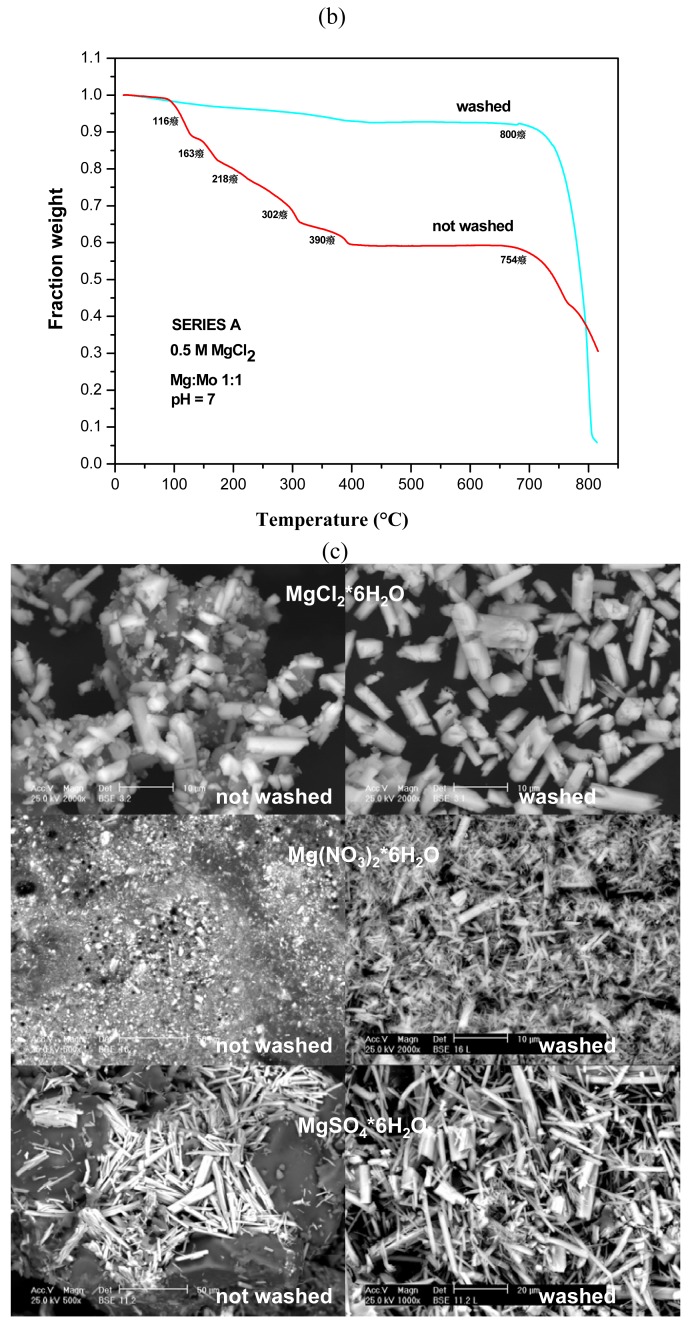
Generator matrix washing effect on (a) X-ray diffraction patters; (b) thermograms and (c) morphology.

The X-ray diffraction patters and thermograms of a typical matrix washed and unwashed are shown in Figure 3a and 3b. The diffractograms show that the washed matrix before irradiation is constituted only by MoO_3_ and the unwashed one by a mixture of MoO_3_ and NH_4_MgCl_3_*6H_2_O. These data match with thermograms of the washed and unwashed matrix which have typical patterns of pure and mixed compounds respectively. Thus, washed matrices before irradiation cause soluble compounds to be removed, mainly those containing ammonium and magnesium in the matrix and conversion of the molybdenum compounds in MoO_3_. It is important to note that this behavior is independent of the type of magnesium salt used in preparing the matrix (Table 1).

The effect of matrix washing on morphology is shown in Figure 3c. The unwashed matrices present a crystalline phase soaked in an amorphous material, and the washed matrices only have the crystalline phase, constituted by rods of different thicknesses and length.

### 2.3. Discussion

The performance of the ^99^Mo/^99m^Tc generators based on magnesium-molybdate compounds depends upon matrix preparation and treatment methods. The latter has to be an insoluble precipitate to avoid ^99^Mo leakage, whilst simultaneously allowing ^99m^Tc release, and have a high Mo content that enables the use of low specific activities of ^99^Mo (2.5 Ci/g) in the generator and a good thermal and radiation stability.

Magnesium molybdate is fairly soluble in water [20-21] and literature has reported obtaining magnesium molybdate precipitates, mainly used in catalysis, applied suitable thermal treatments and magnesium and molybdate concentrations. For example, Yoon *et al.* have reported magnesium molybdates precipitation from Mg(NO_3_)_2_·6H_2_O and (NH_4_)_3_Mo_7_O_24_·4H_2_O solutions and calcining at different temperatures (200–650 °C) [19]. Ozeki *et al.* prepared magnesium molybdate solids by concentrating a mixed solution of 0.1 mol/L sodium molybdate and 4.5 mol/L magnesium chloride [20]. Amber *et al.* obtained MgMoO_4_·H_2_O from 0.1 mol/L Na_2_MoO_4_ and 0.5 mol/L MgCl_2_ solutions at pH 6 treated at 155 °C for 3 days [22].

In this work, insoluble magnesium molybdate compound precipitation was favored by adjusting pH and irradiation, and not by thermal treatment. Under these experimental conditions, magnesium molybdate compounds obtained were mainly polymetalates salts such as xNH_4_MgCl_3_·yMoO_3_, and polymolybdates [NH_4_Mo_5_O_15_(OH)] (see Figure 2 and Table 2). Assuming that compound formation is the result of three steps, firstly the formation of ammonium molybdates according to reaction (1):

(1) $$\text{Mo}{\text{O}}_{3}+2\text{N}{\text{H}}_{4}\text{OH}↔(\text{N}{\text{H}}_{4})\text{Mo}{\text{O}}_{4}+{\text{H}}_{2}\text{O}$$

The molybdate ion generally exists as MoO_4_^2−^ in alkaline or neutral solutions (pH > 6) while polymolybdate ions such as [Mo_7_O_24_]^6−^, [Mo_8_O_26_]^4−^, [Mo_36_O_112_]^8−^ are formed in acid solutions [19,23-27]:

(2) $$\text{xMo}{\text{O}}_{4}^{2-}+\text{y}{\text{H}}^{+}↔\text{M}{\text{o}}_{\text{x}}{\text{O}}_{\text{z}}{\left(\text{OH}\right)}_{8\text{x}-\text{y}-2\text{z}}^{(2\text{x}-\text{y})-}+(\text{y}-4\text{x}-\text{z}){\text{H}}_{2}\text{O}$$

Thus the ammonium molybdate solutions prepared at pH 10 and 7 contain simply MoO_4_^2-^ ions and those at pH 4.5 a mixture of polymolybdate ions where the predominant species is probably the [Mo_7_O_24_]^6−^ ion [18]. In the second step, ammonium molybdates react with magnesium solutions to form magnesium-molybdates solutions. Considering these solutions pH values can vary between 8 and 4.3, the magnesium molybdates can be constituted by MoO_4_^2−^ (pH > 6) or polymolybdates (pH < 6) according to:

(3) $$(\text{N}{\text{H}}_{4})\text{Mo}{\text{O}}_{4}+\text{MgX}↔\text{MgMo}{\text{O}}_{4}+\text{N}{\text{H}}_{4}\text{X}$$

(4) $$\begin{array}{c} {\left(\text{N}{\text{H}}_{4}\right)}_{\left(2\text{x}-\text{y}\right)}\text{M}{\text{o}}_{\text{x}}{\text{O}}_{\text{z}}{\left(\text{OH}\right)}_{8\text{x}-\text{y}-2\text{z}}+(2\text{x}-\text{y})\text{MgX}↔\text{M}{\text{g}}_{\left(2\text{x}-\text{y}\right)}\text{M}{\text{o}}_{\text{x}}{\text{O}}_{\text{z}}{\left(\text{OH}\right)}_{8\text{x}-\text{y}-2\text{z}}+(2\text{x}-\text{y})\text{N}{\text{H}}_{4}\text{X}\\ \text{X}=\text{C}{\text{l}}^{-},\text{N}{\text{O}}_{3}^{-}\;\text{or}\;\text{S}{\text{O}}_{4}^{2-}, \end{array}$$

In the last step, the magnesium molybdates are induced to precipitate by adjusting pH values of the solutions to be between 1.9 and 0.3. At pH < 2, literature has reported the presence of very large polymolybdate species like [Mo_36_O_112_]^8−^ [23,25] or MoO_3_·2H_2_O precipitation [19,28-29]. X-ray diffraction data (see Table 2 and Figure 2) suggests polymetalates formation such as xNH_4_MgCl_3_·yMoO_3_ or polymolybdates according to:

(5) $$\text{MgMo}{\text{O}}_{4}+2\text{N}{\text{H}}_{4}\text{X}+2\text{HCl}↔\text{N}{\text{H}}_{4}\text{MgC}{\text{l}}_{3}\cdot \text{Mo}{\text{O}}_{3}\cdot {\text{H}}_{2}\text{O}+\text{N}{\text{H}}_{4}\text{X}$$

(6) $$\text{uMgMo}{\text{O}}_{4}+\text{vN}{\text{H}}_{4}\text{X}+\text{wHCl}↔\text{u}{\text{NH}}_{4}\text{MgC}{\text{l}}_{3}\cdot {\left(\text{N}{\text{H}}_{4}\right)}_{\left(2\text{x}-\text{y}\right)}\text{M}{\text{o}}_{\text{x}}{\text{O}}_{\text{z}}{\left(\text{OH}\right)}_{8\text{x}-\text{y}-2\text{z}}+(\text{v}-\text{u}-2\text{x}-\text{y})\text{N}{\text{H}}_{4}\text{X}$$

(7) $$\begin{array}{c} \text{M}{\text{g}}_{\left(2\text{x}-\text{y}\right)}{\text{Mo}}_{\text{x}}{\text{O}}_{\text{z}}{\left(\text{OH}\right)}_{8\text{x}-\text{y}-2\text{z}}+\text{vN}{\text{H}}_{4}\text{X}+\text{wHCl}↔(2\text{x}-\text{y})\text{N}{\text{H}}_{4}\text{MgC}{\text{l}}_{3}\cdot {\left(\text{N}{\text{H}}_{4}\right)}_{\left(2\text{x}-\text{y}\right)}\text{M}{\text{o}}_{\text{x}}{\text{O}}_{\text{z}}{\left(\text{OH}\right)}_{8\text{x}-\text{y}-2\text{z}}\\ +\;\text{v}\;-(2\text{x}-\text{y})\text{N}{\text{H}}_{4}\text{X} \end{array}$$

The Cl^−^ ion usually displaces NO_3_^−^ and SO_4_^2−^ ions [30] when magnesium nitrate and sulfate are employed in preparing magnesium molybdates; for that reason ammonium magnesium chlorides (NH_4_MgCl_3_) were present in all the series studied (see Table 2 and Figure 2), however mixtures of NH_4_MgCl_3_ and (NH_4_)_2_Mg(SO_4_)_2_ were also identified in matrices prepared from magnesium sulfates.

An excess of molybdenum favors polymolybdates and formation of amorphous phases whereas a surplus of magnesium the presence of ammonium magnesium salts and crystalline phases. Thus, the crystallinity degree of the compounds contained in the matrix is closely attached to ^99^Mo/^99m^Tc generator performances. For example amorphous matrices presented the best ^99m^Tc elution efficiencies (series B) while the crystalline (series A) presented lower ^99^Mo breakthrough (see Table 1, Figure 1). Assuming that amorphous materials also consist of molybenum oxides or polymolybdates and that the oxides and hydrous oxides of Mo(VI) exhibit cation exchange properties and show little or no anion exchange character even in acid solution [31] and ammonium magnesium salts have no adsorption properties, so the separation mechanism of the ^99^Mo and ^99m^Tc in the generators can be explained by free diffusion of ^99m^TcO_4_^−^ ion inside the matrix because the ^99m^TcO_4_^−^ anion produced in the generator is not adsorbed in the matrix and can be removed from the chromatographic column by elution with isotonic saline solution, leaving the ^99^Mo inside. In accordance with this argument, a crystalline matrix acts as a molecular sieve preventing ^99m^Tc mobility and causing generator efficiency decrease. Whereas a flexible random network (amorphous) increases generator efficiency and radiochemical purity because the matrix is more elastic but simultaneously harder and more resistant to mechanical breakdown and more difficult to dissolve. The low ^99m^Tc eluate radiochemical purities obtained in some generators can be explained by Tc(VII) reduction caused by the presence of insoluble species of polymolybdates, which are strong oxidizing agents [18].

Inorganic materials are susceptible to irradiation-induced amorphization producing particularly volume changes in crystalline or amorphous phases. The main concern with large differential volume changes is that it may affect atomic bonding, local coordination, and the pathways for ion exchange, all of which can impact the release rates of radionuclides [32]. Thus the matrix amorphization caused by its irradiation could be linked to the high ^99^Mo breakthrough obtained in generators for which matrices are mainly formed by amorphous compounds such as the series B and C.

## 3. Experimental

### 3.1. Preparation of Magnesium ^99^Mo-Molybdate Compounds

Magnesium ^99^Mo-molybdate compounds were formed from magnesium and molybdate solutions. The molybdate solutions were prepared from MoO3 natural pellets, previously heated to 650 °C for 1 h and dissolved in 2 mol/L NH_4_OH at a MoO_3_:2NH_4_OH molar ratio [8]. The pH of the formed ammonium molybdates was adjusted by adding 4 mol/L HCl and converted into magnesium molybdate by reacting with magnesium solutions. Magnesium molybdates pH were also adjusted using 4 mol/L HCl. The resulting solids were dried for 2 days using an infrared lamp and crushed in an agate mortar. One portion of magnesium molybdate precipitate was placed on a funnel to be washed using 200 mL of distilled water and the washed and unwashed solids were dried for 1 day at 40 °C in a stove. The dried magnesium molybdate were irradiated for 2 h at a neutron fluence of about 1.61 × 10^13^ n cm^−2^s^−1^ in the Triga Mark III Reactor (Mexico). After irradiation, about 1 g of magnesium ^99^Mo-molybdate (∼4.9 MBq/g) were added into a glass column (12 mm × 70 mm) containing a bed of 1 g acid alumina. The column was finally washed with 20 mL of saline solution [8,17-18]. The magnesium molybdate compounds were synthesized in duplicate at different conditions; where parameters such as magnesium salts and concentrations (MgCl_2_·6H_2_O, Mg(NO_3_)_2_·6H_2_O, MgSO_4_·6H_2_O), Mo:Mg molar ratios, ammonium and magnesium molybdates pH and the addition order of magnesium and molybdenum solutions were evaluated (see Table 2).

### 3.2. Elution of Generators and Eluate Analysis

The generators were eluted with 6 mL of 0.9% NaCl every 24 h for 1 week and the following parameter of the ^99m^Tc eluates were determined: ^99m^Tc elution efficiency, ^99^Mo breakthrough, ^99m^Tc elution profile, ^99m^Tc radiochemical purity, pH eluate and aluminium concentration. The ^99m^Tc elution efficiency and the ^99^Mo breakthrough were calculated from the ^99m^Tc and ^99^Mo activities measured in a CRC-10R Capintec dose calibrator and a GeHp solid state detector (Canberra 7229P) coupled to a PC-multichannel analyzer (ACUSPECT-A, Canberra, Australia). The radiochemical purity of the ^99m^Tc eluate was determined by paper chromatography using 1 CHR (Whatman®) paper as solid phase and 85% methanol as mobile phase. The ^99m^TcO_4_^−^R_f_ was 0.66–0.72. Aluminium and magnesium concentrations in ^99m^Tc eluates were determined by the aluminon and Eriochrome Black T methods [18,33]. The eluate pH values were determined by pH paper.

### 3.3. Gel Characterization

Magnesium-molibdate compounds were characterized by X-ray diffraction (XRD), scanning electron microscopy (SEM), infrared spectrometry, thermogravimetry and neutron activation analysis. The X-ray diffraction patterns were obtained on a Siemens D500 diffractometer for 1 h and scanned from 2.5° to 70° with steps of 0.02°. SEM imaging was performed by Philips SL30. Digital images were obtained at 5,000×, 3,000×, 1,000× and 500× magnifications in randomly selected fields. The infrared measurements were taken on a Nicole Mgna-IR™ spectrometer 550 with the samples pressed in KBr pellets. The thermogravimetric analyses were performed using a Phillips unit at a heating rate of 10°/min under a nitrogen atmosphere [8,18]. Molybdenum and magnesium concentrations were determined by neutron activation. The procedure described in previous works was applied for molybdenum and in the case of magnesium, 50 mg of each magnesium molybdate and MgO, used as reference material, were irradiated in the Triga Mark II reactor at a neutron fluence of about 1.65 × 10^12^ n cm^−2^s^−1^ for 15 s. Magnesium was determined by the 843.4 keV γ-ray of ^27^Mg by means of a HPGe detector at a counting time of 100 s [13].

## 4. Conclusions

The performances of ^99m^Tc generators are strongly related to the chemical composition of the matrix and consequently their preparation conditions. The magnesium molybdate compounds obtained were mainly salts of polymetalates such as NH_4_MgCl_3_·MoO_3_, NH_4_MgSO_4_·MoO_3_ and polymolybdates [Mo_x_O_z_(OH)_8x − y − 2z_^(2x − y)−^] crystallized or amorphous. The type of magnesium salt and the Mg:Mo ratio used in the matrix preparation inhibits or favours polymetalate salts and polymolybdates amorphization. Crystalline NH_4_MgCl_3_·MoO_3_ were preferably obtained from MgCl_2_·6H_2_O solutions while amorphous compounds, probably constituted by polymetalates (see reactions 6 and 7) and unidentified crystalline phases were formed from Mg(NO_3_)_2_·6H_2_O solutions and mixtures of cristalline NH_4_MgCl_3_·MoO_3_ and NH_4_MgSO_4_·MoO_3_ and amorphous phases, also possible formed by polymetalates, were produced from MgSO_4_·6H_2_O solutions. An excess of molybdenum or magnesium during the matrix preparation favors amorphous or crystalline phases formation respectively. The degree of ordering of Mg-Mo compounds defines the ^99m^Tc generators performances: high ^99m^Tc elution efficiencies were obtained from amorphous matrices while lower ^99^Mo breakthrough by crystalline matrices. The free ^99m^TcO_4_^−^ diffusion is proposed as separation mechanism of the ^99^Mo and ^99m^Tc in the generators considering that polymetalates act as cation exchanges and the ^99m^TcO_4_^−^ anion produced in the generator is not adsorbed in the matrix.

^99^Mo/^99m^Tc generator production based on magnesium-^99^Mo molybdate compounds allow reduction of preparation time and eliminates the use of specialized installations. The best generator performances were attained using matrices prepared from 0.1 mol/L MgCl_2_·6H_2_O solutions, ammonium molybdate solutions at pH 7 and at a Mg:Mo molar ratio of 1:1.

## Figures and Tables

**Table 1:** Performances of ^99^Mo/^99m^Tc generators based on magnesium ^99^Mo-molybdate compounds.

**Series**	**[Mg^2+^]**	**Mg:Mo**	**pH ammonium molybdates**	**Eluate color**	**Eluate Volume (mL)**	**^99^Mo Breakthrough (%)**	**^99m^Tc elution efficiency (%)**	**^99m^TcO_4_^−^ (%)**	**Al^3+^ 10ppm**	**Mg^2+^10ppm**	**pH eluate**	**washed**	**Mo %**	**Mg %**	**Crystalline phases**
**A1**	0.5 M	0.64:1	7	colorless	3.5	0.022	72.3	68-82	<	<	3.8–4.4	no	61.44	11.00	MoO_3_, NH_4_ MgCl_3_*6H_2_O
**A2**	0.5 M	0.93:1	7	colorless	2	0.014	17.7	73	<	<	4.5–5	no	49.44	8.89	MoO_3_, NH4 MgCl_3_*6H_2_O
**A2w**	0.5 M	0.93:1	7	colorless	2.5	0.071	31.1	75	<	<	3.3–5	yes	47.98	2.52	MoO_3_
**A3**	0.5 M	1.08:1	7	colorless	6	0	48	89	<	<	3.2	no	67.4	17.11	MoO_3_, NH4 MgCl_3_*6H_2_O
**A3w**	0.5 M	1.08:1	7	colorless	3.5	0.2	49.8	50	<	<	3.2–4.2	yes	55.97	2.51	MoO_3_
**A4**	0.5 M	1.18:1	7	colorless	2	0.025	24.2	84–94	<	<	4.4–5.7	no	75.83	11.55	MoO_3_, NH_4_ MgCl_3_*6H_2_O
**A4w**	0.5 M	1.18:1	7	colorless	2.5	0.0026	43.2	37–77	<	<	4.5–5	yes	47.20	4.29	MoO_3_
**A5**	1 M	2:1	7	colorless	2	1.37	29	93	<	>	2.8–3.7	no	15.8	21.35	NH_4_ MgCl_3_*6H_2_O, NH_3_(MoO_3_)_3_, NH_4_Cl
**A5w**	1 M	2:1	7	colorless	3	0.035	34	93	<	<	2.2–3.1	yes	28.03	0.69	MoO_3_
**A6**	05 M	2:1	7	colorless	3	0.85	52	90	<	<	4.8–5.5	no	11.28	22.27	NH_4_ MgCl_3_*6H_2_O, NH_3_(MoO_3_)_3_
**A6w**	05 M	2:1	7	colorless	3	0.06	16	98	<	<	4.3–6.8	yes	58.81	1.62	MoO_3_
**A7**	0.5 M	1:1	7	colorless	2.5	4.8	29.3	98	<	>	4–4.2	no	28.05	20.71	NH_4_ MgCl_3_*6H_2_O, NH_3_(MoO_3_)_3_, NH_4_Mo_5_O_15_(OH)*2H_2_O
**A8**	0.5 M	1:2	7	colorless	2.3	0	20	84	<	<	5.2–7.2	no	47.5	18.55	NH_4_ MgCl_3_*6H_2_O, NH_3_(MoO_3_)_3_, NH_4_Mo_5_O_15_(OH)*2H_2_O
**A9**	0.1 M	1:2	7	colorless	3.5	7.07	10	92	<	>	6.8–7	no	18.16	10.8	NH_4_ MgCl_3_*6H_2_O, NH_3_(MoO_3_)_3_, MoO_3_
**A10**	0.5 M	2:1	4.5	colorless	2.8	0.1	69	95	<	<	4.0–5.2	no	9.08	19.35	NH_4_ MgCl_3_*6H_2_O, NH_4_Cl, NH_3_(MoO_3_)_3_
**A11**	0.5 M	1:2	4.5	colorless	2.5	1.1	37	20	<	<	4.3–5.5	no	49.71	9.22	NH_3_(MoO_3_)_3_, MoO_3_, NH_4_Mo_5_O_15_(OH) 2H_2_O, NH_4_ MgCl_3_*6H_2_O
**A11w**	0.5M	1:2	4.5	colorless	3	0	20	60	<	<	5.9–6.4	yes	54.85	0.59	MoO_3_,
**A12**	0.5M	1:1	4.5	colorless	3	0.95	26.2	96	<	<	2.4–4	no	68.54		MoO_3_, MoO_3_*H_2_O
**B1**	0.1M	0.2:1	7	colorless	2	4.24	83.9	87	<	<	2.7–3	no	27.19	5.03	amorphous, unidentified phases
**B2**	0.1M	0.2:1	7	yellow	2.5	1.9	87.8	91	<	<	1.7–2.6	no	19.97	3.63	amorphous, unidentified phases
**B3**	0.1M	0.2:1	4.5	colorless	2	1.16	77	79	<	<	2.2–4	no	21.31	2.45	amorphous, unidentified phases
**B4**	0.1M	0.2:1	4.5	yellow	2	10.74	70.1	85	<	<	2–3	no	26.05	2.89	amorphous, unidentified phases
**B5**	0.1M	0.2:1	10	yellow	2.5	1.8	81.3	89	<	<	2–3.6	no	26.91	2.93	amorphous, unidentified phases
**B5w**	0.1 M	0.2:1	10	yellow	6	0.74	76.84	83	<	<	3.8–2.6	yes			
**B6**	0.1 M	0.2:1	10	yellow	2.5	1.6	79.3	90	<	<	2.3–5.3	no	25.83	1.56	amorphous, unidentified phases
**B6w**	0.1 M	0.2:1	10	yellow	2.5	4.17	42.7	78	<	>	2.9–3.7	yes			MoO_3_, NH_3_(MoO_3_)_3_
**B7**	1 M	1:1	7	colorless	5	0	79.7	52–92	<	>	3.4–4.2	no	42.3	33.37	NH_4_ MgCl_3_*6H_2_O, NH_3_(MoO_3_)_3_
**B7w**	1 M	1:1	7	colorless	3	0.39	55.4	98	<	<	4.6–5	yes	64.9	0.88	MoO_3_
**B8**	0.1 M	1:2	4.5	colorless	3	0.035	53.5	96–90	<	<	5–6.3	no	47.65		MoO_3_, NH_4_ MgCl_3_*6H_2_O, (NH_4_)_2_Mo_3_O_10_
**B9**	0.1 M	2:1	4.5	colorless	2	1.23	57.5	99	<	<	4.3–4.8	yes		4.12	amorphous, unidentified phases, NH_4_ MgCl_3_*6H_2_O
**C1**	1 M	2:1	4.5	yellow	2.5	0.82	29	82	<	>	1–1.6	no	18.16	35.72	NH_4_ MgCl_3_*6H_2_O, (NH_4_)_2_Mg(SO_4_)_2_*6H_2_O
**C2**	0.1 M	2:1	4.5	colorless	4	0.19	50	89–96	<	<	1.9–3.6	no	15.7	19.93	NH_4_ MgCl_3_*6H_2_O, (NH_4_)_2_Mg(SO_4_)_2_*6H_2_O, NH_4_Cl,
**C2w**	0.1 M	2:1	4.5	colorless	2	2.2	20	89–93	<	<	3.9–4.4	yes	74.28	0.57	MoO_3_
**C3**	0.05 M	2:1	4.5	yellow-clear	2	3.89	56.5	90	<	>	3.1–3.4	no	7.11	24.2	NH_4_MgCl_3_ 6H_2_O
**C3w**	0.05 M	2:1	4.5	Blue precipited	1.5	3.8	50	81–94	≤	<	2.5–1.7	yes	34.16	2.84	amorphous, unidentified phases
**C4**	1 M	1:1	7	clear precipited	2.	5.59	40.6	78	<	>	1.9–2	no	11.34	10.08	NH_4_ MgCl_3_*6H_2_O, (NH_4_)_2_Mg(SO_4_)_2_ 6H_2_O, amorphous
**C4w**	1 M	1:1	7	colorless	3	0.72	15	81–95	<	<	3.4–4.2	yes	69.53		MoO_3_

**Table 2:** Preparation conditions of magnesium molybdate compounds.

**Series**	**pH Ammonium molybdates**	**Mg:Mo**	[MgCl_2__*_6H_2_O] **mol/L**	[MgCl_2__*_6H_2_O] **pH**	**pH Magnesium molybdate**	**Addition order**
A1	7	0.64:1	0.5	0.72	0.05	Mg(NO_3_)_2_→ [MoO_4_^2−^]
A2	7	0.93:1	0.5	0.72	0.49	Mg(NO_3_)_2_→ [MoO_4_^2−^]
A3	7	1.08:1	0.5	5.9	0.3	Mg(NO_3_)_2_→ [MoO_4_^2−^]
A4	7	1.18:1	0.5	0.1	0.5	Mg(NO_3_)_2_→ [MoO_4_^2−^]
A5	7	2:1	1	5.9	1.2	Mg(NO_3_)_2_→ [MoO_4_^2−^]
A6	7	2:1	0.5	0.7	0.4	Mg(NO_3_)_2_→ [MoO_4_^2−^]
A7	7	1:1	0.5	0.7	0.5	Mg(NO_3_)_2_→ [MoO_4_^2−^]
A8	7	1:2	0.5	0.7	0.6	Mg(NO_3_)_2_→ [MoO_4_^2−^]
A9	7	1:2	0.1	0.7	0.7	Mg(NO_3_)_2_→ [MoO_4_^2−^]
A10	4.5	2:1	0.5	0.7	0.6	Mg(NO_3_)_2_→ [MoO_4_^2−^]
A11	4.5	1:2	0.5	0.7	1.8	Mg(NO_3_)_2_→ [MoO_4_^2−^]
A12	4.5	1:1	0.5	0.7	1.6	Mg(NO_3_)_2_→ [MoO_4_^2−^]
**Series**	**pH Ammonium molybdates**	**Mg:Mo**	[Mg(NO_3_)_2_*6H_2_O] **mol/L**	**pH** [Mg(NO_3_)_2_*6H_2_O]	**pH Magnesium molybdate**	**Addition order**
B1	7	0.2:1	0.1	5.5	1.1	Mg(NO_3_)_2_→ [MoO_4_^2−^]
B2	7	0.2:1	0.1	5.5	0.7	[MoO_4_^2−^] → Mg(NO_3_)_2_
B3	4.5	0.2:1	0.1	5.5	0.9	Mg(NO_3_)_2_→ [MoO_4_^2−^]
B4	4.5	0.2:1	0.1	5.5	0.9	[MoO_4_^2−^] → Mg(NO_3_)_2_
B5	10	0.2:1	0.1	5.5	1.0	Mg(NO_3_)_2_→ [MoO_4_^2−^]
B6	10	0.2:1	0.1	5.5	0.9	[MoO_4_^2−^] → Mg(NO_3_)_2_
B7	7	1:1	1	5.5	0.1	Mg(NO_3_)_2_→ [MoO_4_^2−^]
B8	4.5	1:2	0.1M	5.5	1.0	Mg(NO_3_)_2_→ [MoO_4_^2−^]
B9	4.5	2:1	0.1M	5.5	1.9	Mg(NO_3_)_2_→ [MoO_4_^2−^]
**Series**	**pH Ammonium molybdates**	**Mg:Mo**	[MgSO_4_*6H_2_O] **mol/L**	**pH** [MgSO_4_*6H_2_O]	**pH Magnesium molybdate**	**Addition order**
C1	4.5	2:1	1	5.4	1.0	Mg(NO_3_)_2_→ [MoO_4_^2−^]
C2	4.5	2:1	0.1	5.4	0.6	Mg(NO_3_)_2_→ [MoO_4_^2−^]
C3	4.5	2:1	0.05	5.4	0.9	Mg(NO_3_)_2_→ [MoO_4_^2−^]
C4	7	1:1	1	5.4	1.0	Mg(NO_3_)_2_→ [MoO_4_^2−^]
